# Combined Treatment of Adipose Derived-Mesenchymal Stem Cells and Pregabalin Is Superior to Monotherapy for the Treatment of Neuropathic Pain in Rats

**DOI:** 10.1155/2021/8847110

**Published:** 2021-02-15

**Authors:** Shimaa Mohammad Yousof, Doaa Attia ElSayed, Amani A. El-Baz, Hanaa S. Sallam, Faten Abbas

**Affiliations:** ^1^Department of Physiology, Faculty of Medicine, Suez Canal University, Ismailia, Egypt; ^2^Department of Physiology, Faculty of Medicine, King Abdulaziz University, Rabigh, Saudi Arabia; ^3^Endocrinology Division, Internal Medicine Department, University of Texas Medical Branch, Texas, USA

## Abstract

**Aims:**

Neuropathic pain following nerve injury does not respond well to most available pharmacological remedies. We aimed to compare the outcome of the addition of adipose-derived mesenchymal stem cells (ADMSCs) to pregabalin for neuropathic pain treatment.

**Methods:**

Adult female albino rats (*n* = 100) were randomized to receive traumatic sciatic nerve injury or sham. Animals were then randomized to ADMSC treatment with or without pregabalin. We conducted a battery of neurobehavioral and electrophysiological to assess neuropathic pain. Following sacrifice, we evaluated the histological changes and gene expression of brain-derived neurotrophic factor (BDNF) in the sciatic nerve. Serum and sciatic nerve tissue pro- and inflammatory cytokine levels were also assessed.

**Results:**

(1) All treatments significantly improved thermal withdrawal latency, sciatic nerve conduction velocity, and proinflammatory cytokine levels in injured animals, with no significant effect of the combined treatments compared to pregabalin monotherapy (*p* < 0.05 each). (2) Combined treatment significantly improved medial gastrocnemius electromyographic amplitude and sciatic function index compared to pregabalin monotherapy (*p* < 0.05 each). (3) Combined treatment significantly increased the BDNF expression, decreased anti-inflammatory cytokine (*p* < 0.05 each), and restored the structural nerve damage, compared to pregabalin monotherapy.

**Conclusions:**

Combined treatment is associated with greater improvement of the sciatic nerve structure and function. Further studies are warranted to study the mechanism of action of the combined treatment to improve neuropathic pain.

## 1. Introduction

Neuropathic pain is responsible for about 35% of the painful conditions that affect the population [1]. Neuropathic pain is more prevalent, frequent, and intense in females with diabetes [2, 3]. Management of neuropathic pain is mainly conservative and symptomatic since the cause of the pain can rarely be treated [4]. Neuropathic pain is considered one of the clinical problems that are difficult to treat, impacting the individual's physical, psychological, and social welfare. The burden also extends to the health services and facilities treating patients suffering from this chronic, debilitating condition [5, 6].

The European Federation of Neurological Societies recommends pregabalin as the first choice for the treatment of diabetic or central neuropathic pain [4]. The neuropathic pain response to pregabalin can be enhanced by combining it with a tricyclic antidepressant or opioid [5, 7]. However, the adverse effects of combined treatment remain substantial [8]. New combination approaches to improve antinociception with fewer side effects should be considered and investigated [9].

Adipose-derived mesenchymal stem cells (ADMSCs) could modulate the nervous system's injured environment and promote repair since they secrete both anti-inflammatory and antiapoptotic molecules, as well as trophic factors to support axonal growth, immunomodulation, angiogenesis, remyelination, and protection from apoptotic cell death [10, 11]. ADMSCs provide an excellent therapeutic potential in neuropathic pain as they secrete “secretomes,” including growth factors (e.g., nerve and vascular endothelial growth factors). ADMSCs have advantages over other types of MSCs, as it requires minimally invasive techniques yet yields large numbers of MSCs. In addition, ADMSCs have high immunomodulatory properties and low immunogenicity, an advantage for treating neuroinflammatory diseases, such as neuropathic pain [12].

In brief, there is a strong need to find practical therapeutic approaches for neuropathic pain with lesser side effects and better outcomes. Combining two lines of therapy acting via different mechanistic pathways could work synergistically to improve neuropathic pain. In this study, we explored the outcome of the sole use of pregabalin or ADMSCs, versus a combination of both in an animal model of neuropathic pain.

## 2. Materials and Methods

### 2.1. Animals

Adult female Albino rats (*n* = 150, 250-300 gm) purchased from the Ophthalmology Research Institute in Giza were housed in the animal house of the Physiology Department, Faculty of Medicine, Suez Canal University, in cages (5 each) at controlled room temperature and husbandry conditions. The animals had free access to water and a standard rat chow diet while observing a 12-hour dark/light cycle. This study was approved by the Ethical & the Scientific Research Committee of the Faculty of Medicine, Suez Canal University, in January 2015. Our methodology is following the principles and standards for reporting animal experiments. We strictly followed the ethical guidelines for investigations of experimental pain in conscious animals, according to the Committee for Research and Ethical Issues of the International Association for the Study of Pain. Every effort was made to minimize the pain and shorten its duration.

Following 1-week acclimatization, rats were randomized to undergo sham (*n* = 30) or sciatic nerve crush surgery (*n* = 120). One week after surgery, animals were randomized to receive a single dose treatment with phosphate-buffered saline (PBS; Sigma-Aldrich-intravenous (IV)—injury group) or ADMSCs (1 × 10^6^ cells suspended in PBS-IV—ADMSCs group), pregabalin (25 mg/kg—intraperitoneal (IP) —pregabalin group), or a combined treatment of ADMSCs (1 × 10^6^ cells IV)+pregabalin (25 mg/kg IP—combined group**)**. Assessment of hyperalgesia was done on days days 1, 7, 14, and 21 after treatment. Animals were randomized to be sacrificed on days 1, 7, and 21 posttreatment (*n* = 10 of each group). Other physiological assessments were made before sacrifice on day 21. Tissues and serum were collected for further assessments (Figure S1 shows the study design workflow chart).

Sciatic nerve crush injury was inflicted as previously described [13]. Briefly, the left sciatic nerve was exposed then crushed with smooth-tipped forceps (5 mm) for 30 seconds under general anesthesia by sodium thiopental (50 mg/kg-IP). In sham surgery, the sciatic nerve was exposed but not crushed. The surgical incision was sutured, and animals were allowed to recover for one week.

### 2.2. Isolation of Human ADMSCs

All patients donating the adipose tissue as a source of ADMSCs provided written informed consent. We chose to use human-derived ADMSCs as lipoaspirate donations were readily available for research from healthy females (age 36–45 years—*n* = 5) undergoing abdominoplasty at the Suez Canal University Teaching Hospital. The lipoaspirate was first washed with PBS (Sigma-Aldrich) containing 10% antibiotic–antimycotic mixture and then minced with a blade into small fragments before being immersed in a solution of 0.2% collagenase type I (Sigma-Aldrich) in PBS for 40 minutes at 37°C under gentle stirring. The digested tissue was filtered using a 100 *μ*m filter mesh (Sigma-Aldrich) and centrifuged at 1250 rpm for 5 minutes at 20°C, after which the supernatant was eliminated. The cells were seeded in 25 cm^2^ culture flasks with a basal medium composed of Dulbecco's Modified Eagle's Medium (DMEM) (Sigma-Aldrich), 10% fetal bovine serum, and 1% antibiotic–antimycotic, until the confluence was 80%. Subsequently, the cells were trypsinized and collected in PBS, ready to assess stem cell identification and transplantation.

### 2.3. Immunomagnetic Separation of ADMSCs

We used the immunomagnetic cell separation technique as recommended [14]. Detached cells underwent immunomagnetic separation according to the manufacturer's instructions (Miltenyi Biotech) for the surface antigens CD105, CD34, and CD45 [15]. Briefly, the cell suspension was centrifuged, and the pellet was resuspended in buffer. According to the cell count, the fragment crystallizable region receptor blocking reagent was added as well as microbeads for the surface antigen tested (either CD105, CD34, or CD45). Cells were mixed and refrigerated for 30 minutes before buffer was added and centrifuged with the pellet resuspended in buffer. The magnetic-associated cell separation column (MACS) separation unit (magnet) was placed to the multistand of MiniMACS. The magnet separation column was placed in the MACS separation unit, and a sequence of repeated washing of all labeled cells was done. Cells were pelleted by low-speed centrifugation (1500 rpm for 5 minutes), washed, and cell suspension centrifuged. The supernatant was then aspirated completely and the cells were counted. Stem cells were positive for CD105 and negative for CD34 and CD45, consistent with the surface marker profile of ADMSC.

### 2.4. Functional Assessments

#### 2.4.1. Thermal Hyperalgesia

A measurement of nociceptive behaviors was tested on days 1 and 7 postinjury and 1, 7, 14, and 21 posttreatment using the hotplate method. Animals were acclimatized to the test environment by placing them in a glass jar on a hotplate (while turned off) for 15 minutes prior to the test. On the assessment day, the hotplate was set to give each paw a heat source, and the thermal withdrawal latency (in seconds) was measured. The heat source was set at an intensity of 52-53°C, with a cut-off time of 40 seconds, to prevent thermal injury to the footpad. Each hind paw was tested three times. Withdrawal latency measurements from each hind paw were averaged, and the total withdrawal latency was calculated [16].

#### 2.4.2. Walking Track Analysis for Motor Function Evaluation

To assess the effect of nerve repair on the motor function, animals underwent walking track analysis at day 21 posttreatment (*n* = 30). Following acclimatization in the walking track analysis apparatus, each rat's forelimbs and hind limbs were dipped in methylene blue to mark its footprints. The apparatus composed of an open field (60 cm × 60 cm × 40 cm) illuminated by a light, in which a runway (4.5 cm wide, 42 cm long, borders 12 cm high) was arranged to lead out into a dark wooden box (20 cm × 17 cm × 10 cm). Rats were allowed to walk across a white sheet towards a custom-built dark escape box, leaving a trace of their paw prints on the sheet. Runs in which the rats made stops or obvious decelerations observed by the examiner were excluded from the analysis. Data analysis was done by comparing four measurements between the injured and the normal side. Measurements taken from footprints include print length; distance from the heel to the third toe, toe spread; distance from the first to the fifth toe, intermediate toe spread; and distance from the second to the fourth toe. These measurements were used to calculate the sciatic function index (SFI) [17, 18]. An SFI of 0 indicates normal, and -100 indicates total impairment. Measurements, including print length, on both the experimental and the normal limbs were used to calculate the SFI as follows:

(1) $$\begin{array}{c} \text{SFI}=-38.3\left\{\text{EPL}-\text{NPL}/\text{NPL}\right\}+109.5\left\{\text{ETS}-\text{NTS}/\text{NTS}\right\}+13.3\left\{\text{EIT}-\text{NIT}/\text{NIT}\right\}-8.8 \end{array}$$

*E* stands for experimental and *N* for normal. PL: Print Length; TS: toe spread IT: intermediate toe spread.

### 2.5. Electrophysiological Assessments

Animals were randomized to be sacrificed on days 1, 7, or 21 posttreatment (*n* = 30 of each group–Figure S1) under general anesthesia with sodium pentobarbital (60 mg/kg, IP, 0.1 mL/10 g) followed by cervical dislocation.

#### 2.5.1. Electromyography (EMG)

Prior to the sacrifice on day 21 posttreatment, stimulating hooked platinum electrodes were placed around the sciatic nerve 5 mm proximal to the crushed site under general anesthesia by sodium thiopental (50 mg/kg—IP—*n* = 30). The electrical current application started with a monophasic, single, square pulse with a duration of 1 ms and an intensity of 10 *μ*A produced by an electric stimulator (EMG100C, Biopac Systems, Inc., USA). The intensity was gradually increased until the supramaximal stimulation that ensured maximal amplitude was reached (1 mA). After that, the recorded signals were digitally converted, with an MP 150 Biopac System, into data. The recording electrodes were placed in the medial gastrocnemius muscle through a percutaneous puncture, ipsilaterally to the surgical procedure. The positive electrode was applied in the muscle origin, the negative electrode in the muscle insertion (back of the knee), and the ground electrode in the rat's tail. The amplitude was calculated from the baseline to the maximal peak [19].

#### 2.5.2. Nerve Conduction Velocity

Following the sacrifice on day 21, posttreatment left sciatic nerves were dissected from the spinal emergence to the knee and stored in Ringer's solution (*n* = 30). Nerve stimulation and recording were done using the Biopac mp150 Data Acquisition System. The acrylic nerve stimulation holder (8 × 4.5 × 2.5 cm) contained chambers filled with Ringer's solution. A segment of the nerve (30 to 35 mm) was placed in the chamber to allow for good electrical contact with the measuring and stimulating electrodes. Ringer's solution temperature was monitored and maintained at room temperature (approximately 20–23°C). A stimulus was applied at 50 millisecond duration, with an intensity set at 10 *μ*A. Nerve conduction velocity was measured by dividing the distance between the stimulating and recording electrodes by the time elapsed between the initiation of the stimulus and the time when the action potential occurred.

### 2.6. Brain-Derived Neurotrophic Factor (BDNF) Gene Expression in the Sciatic Nerve

Following the sacrifice on day 21 posttreatment, the dissected sciatic nerves were subjected to RNA extraction. Reverse transcription was done with random hexamer primers by Superscript III First-Strand Synthesis (Qiagen company). RT-PCR for BDNF mRNA from the sciatic nerves was performed, as previously described [19]. The reverse-transcribed RNA was amplified using the Prism7000 Sequence Detection System (Applied Biosystems) with QuantiTect SYBR Green PCR Kits (QIAGEN). The following primers were used for real-time PCR amplification: BDNF (forward: 5′-CGGCGCCCATGAAAGAAGTA-3′; reverse: 5′-AGACCTCTCGAACCTGCCCT-3′). 18S rRNA (forward: 5′-TTAACGAGGATCCATTGGAG-3′; reverse: 5′-GGCCTGCTTTGAACACTCTA-3′) was used as an endogenous control to obtain *Δ*Ct. Fold change was expressed by 2^-*ΔΔ*Ct^.

### 2.7. Biochemical Analysis: Cytokine Evaluation

Following the sacrifice on days 1 and 7 posttreatment, the ipsilateral injured sciatic nerve, proximal to the trifurcation (about 1 cm), was removed under a dissecting microscope and immediately frozen in liquid nitrogen. It was then stored at −80°C for further assessment of inflammatory markers (interleukin 1*β* “IL-1*β*”; (interleukin 10 “IL-10”) and tumor necrosis factor-alpha (TNF-*α*). The nerve samples were homogenized in 0.4 mL of ice-cold PBS containing a protease inhibitor cocktail (Sigma-Aldrich) and centrifuged at 10,000 g for 15 minutes [15]. The supernatant was used to measure rat IL-1*β*, IL-10, TNF-*α* levels, and total protein content. IL-1*β* and IL-10 protein contents were determined by the enzyme-linked immunosorbent assay (ELISA), using ultrasensitive ELISA kits according to the manufacturer's instruction (US assay pro company, The Assay Max Interleukin-10 catalog number: E13010-1, IL-1 beta Catalog No. EI2200-1, and TNF-*α* Catalog No. ET2010-1). Cytokine concentrations were determined by interpolation with standard curves assayed on individual plates normalized to the protein content in each sample.

### 2.8. Histopathological Assessment

H&E Following the sacrifice on day 21 posttreatment, hematoxylin and eosin (H&E) stains of nerve sections (8 m) were deparaffinized, then rehydrated and stained for 2 minutes in Ehrlich's H&E stain (Sigma-Aldrich). The sections were dipped in ammonia water, rinsed with tap water, and then stained in eosin for 5 minutes, followed by three dips in 95% ethanol [20]. All slides were captured with a high-power field of ×400 and intermediate power of 100×s. The histological examination was done blindly by an expert investigator.

### 2.9. Data Analysis

All numerical data were analyzed with SPSS statistical software version 25. Data were presented as mean ± SD. One-way analysis of variance (ANOVA) was used for comparing the means of a variable in the five groups for all studied variables, except for the thermal withdrawal latency, where a repeated measure ANOVA was used to compare the means in all five groups, overtime. In case a statistically significant difference is detected by ANOVA, a posthoc test (Bonferroni test) was used to analyze the results further. A *p* value <0.05 was considered statistically significant. Pearson's correlation was used to test correlations.

## 3. Results

### 3.1. Functional Assessments

**Figure 1:**
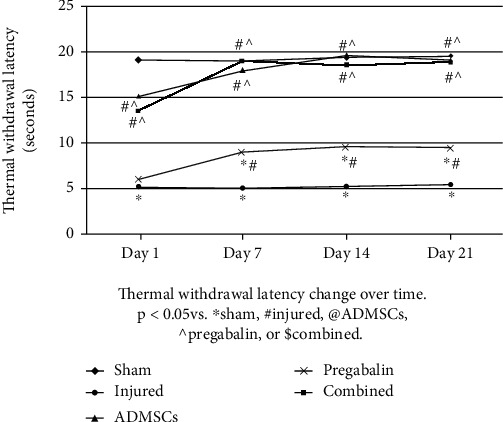
Thermal withdrawal latency changes over time. All animals in the injured group showed a significant decrease in thermal withdrawal latency along the course of the experiment compared to the sham group. All treatments improved thermal withdrawal latency over time (*p* < 0.05 vs. ^∗^sham, #injured, @adipose tissue-derived mesenchymal stem cells (ADMSCs), ^pregabalin, or $combined). There was no statistical difference between combined and ADMSC treatments (*p* > 0.05).

Thermal withdrawal latency (seconds) was assessed on day 1 postinjury to confirm the establishment of neuropathic pain. Repeated measure ANOVA for thermal withdrawal latency on days 7, 14 and 21 showing significant differences among groups (*p* < 0.01). Posthoc Bonferroni test revealed that the injured group showed significant decrease in the thermal withdrawal latency compared to the sham group (*p* < 0.01) over time. All treated groups showed significant increase in the thermal withdrawal latency compared to the injured group throughout the experiment (*p* < 0.01). The ADMSCs and combined groups showed insignificant difference when compared to each other (*p* > 0.05). The combined group showed significant increase in the thermal withdrawal latency compared to the pregabalin group throughout the course of treatment (*p* < 0.01). Interestingly, there was no statistical difference between combined and ADMSC treatments (*p* > 0.05-Figure 1).

**Figure 2:**
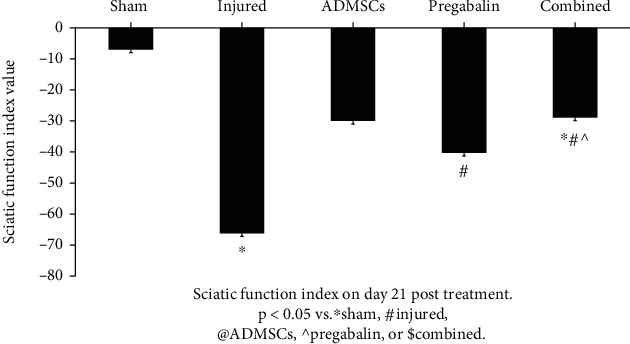
Sciatic function index (SFI) on day 21 posttreatment. The injured group showed deterioration of the SFI compared to the sham group. All treated groups showed significant improvement in the SFI (*p* < 0.05 vs. ^∗^sham, #injured, @adipose tissue-derived mesenchymal stem cells (ADMSCs), ^pregabalin, or $combined). The combined treatment showed a highly significant improvement in SFI compared to pregabalin, but not ADMSCs alone (*p* > 0.05).

The injured group showed deterioration of the SFI compared to the sham group (−66.2 ± 4 vs. -7.0 ± 2 for injury and sham, respectively-*p* < 0.05). All treated groups showed significant improvement in the SFI (−28.9 ± 3, −30.0 ± 3, and −40.3 ± 5, for combined, ADMSCs, and pregabalin, respectively-*p* < 0.05). Interestingly, the combined treatment showed a highly significant improvement in SFI compared to pregabalin (*p* < 0.01), but not ADMSC alone (*p* > 0.05-Figure 2).

### 3.2. Electrophysiological Assessments

**Figure 3:**
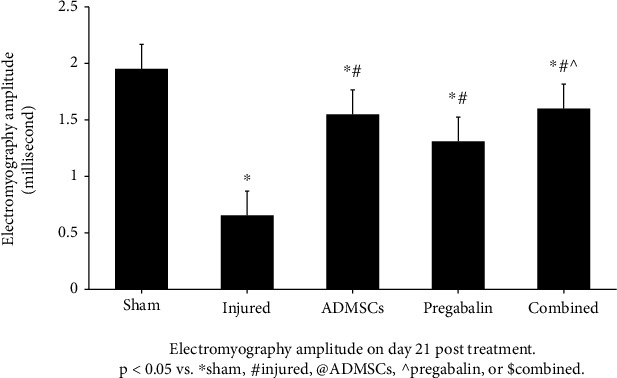
Electromyography amplitude on day 21 posttreatment. The injured group showed a significant decrease in EMG amplitude compared to the sham group. All treated groups showed an increase in EMG amplitude (*p* < 0.05 vs. ^∗^sham, #injured, @adipose tissue-derived mesenchymal stem cells (ADMSCs), ^pregabalin, or $combined). The combined treatment showed a significant improvement in EMG amplitude compared to pregabalin (*p* < 0.05), but not to ADMSCs alone (*p* > 0.05).

The injured group showed a significant decrease in EMG amplitude (mV) compared to the sham group (0.7 ± 0.2 vs. 2 ± 0.2 for injury and sham, respectively-*p* < 0.05). All treated groups showed an increase in EMG amplitude (1.6 ± 0.3, 1.5 ± 0.2, and 1.3 ± 0.2 for combined, ADMSCs, and pregabalin, respectively-*p* < 0.05). Interestingly, the combined treatment showed a significant improvement in EMG amplitude compared to pregabalin (*p* < 0.05), but not to ADMSC alone (*p* > 0.05-Figure 3).

**Figure 4:**
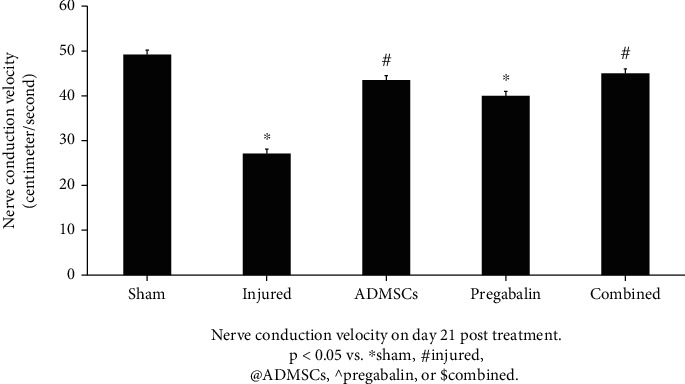
Nerve conduction velocity on day 21 posttreatment. The injured group showed a significant decrease in nerve conduction velocity (cm/seconds) compared to the sham group. All treated groups showed an increase in the nerve conduction velocity (*p* < 0.05 vs. ^∗^sham, #injured, @adipose tissue-derived mesenchymal stem cells (ADMSCs), ^pregabalin, or $combined). The combined treatment was not significantly different than ADMSCs or pregabalin alone (*p* > 0.05).

The injured group showed a significant decrease in nerve conduction velocity (cm/seconds) compared to the sham group (27.1 ± 2.2 vs. 49.2 ± 3.3 for injury and sham, respectively-*p* < 0.05). All treated groups showed an increase in the nerve conduction velocity (45 ± 4.3, 40 ± 5.1, and 43.5 ± 7.1 for combined, ADMSCs, and pregabalin, respectively-*p* < 0.05). Interestingly, the combined treatment was not significantly different than ADMSCs or pregabalin alone (*p* > 0.05-Figure 4).

### 3.3. Brain-Derived Neurotrophic Factor (BDNF) Gene Expression in the Sciatic Nerve

**Figure 5:**
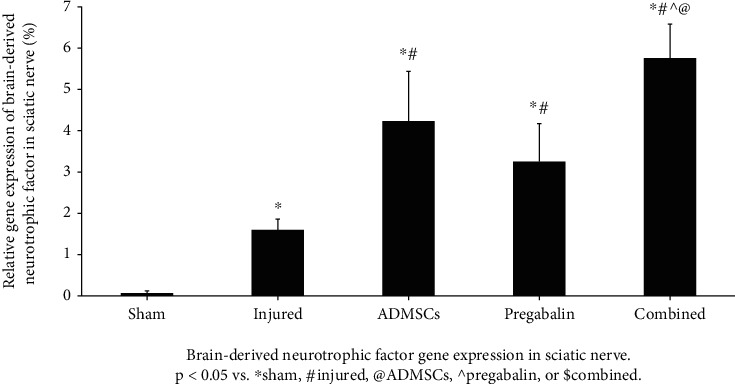
Brain-derived neurotrophic factor gene expression in the sciatic nerve. The injured group showed a significant increase in the BDNF expression compared to the sham group. All treated groups showed a higher BDNF expression (*p* < 0.05 vs. ^∗^sham, #injured, @adipose tissue-derived mesenchymal stem cells (ADMSCs), ^pregabalin, or $combined). The combined treatment showed a significant improvement compared to ADMSCs or pregabalin alone (*p* < 0.001 and 0.0001, respectively).

The injured group showed a significant increase in the BDNF expression compared to the sham group (1.6 ± 0.26 vs. 0.06 ± 0.6 for injury and sham, respectively-*p* < 0.05). All treated groups showed a higher BDNF expression (5.8 ± 0.82, 4.2 ± 1.2, and 3.3 ± 0.92, for combined, ADMSCs, and pregabalin, respectively—*p* < 0.05). Interestingly, the combined treatment showed a significant improvement compared to ADMSCs or pregabalin alone (*p* < 0.001 and 0.0001, respectively-Figure 5).

### 3.4. Biochemical Analysis: Cytokine Evaluation

Table S1 shows the serum and sciatic nerve tissue levels of inflammatory markers on days 1 and 7 posttreatment. ADMSCs or combined treatment almost normalized tissue TNF-*α*, the proinflammatory cytokine, while increasing IL-10, the anti-inflammatory cytokine, compared to the injured group.

### 3.5. Histopathological Assessment

The injured group showed histopathological characteristics of nerve injury compared to the sham group in the form of disorganized axons with marked vacuolar degeneration, large empty vacuoles in between axons, a thickened wall of a blood vessel, and an increase in inflammatory cellular infiltration (Figure S2A, B). Additionally, most axons were thinned out, while some collapsed and were transformed into a pink hyaline material. ADMSCs moderately restored the axons' regular arrangement and increased the thickness and regularity of the nerve (Figure S2C). Pregabalin mildly restored the regular arrangement of the axons and the regularity of the nerve (Figure S2D). Interestingly, the combined treatment strongly restored the regular arrangement of the axons and the regularity of the nerve (Figure S2E).

### 3.6. Correlations

On day 1 postinjury, there was a robust positive correlation between SFI and the levels of IL1-*β* in the sciatic nerve (*r*^2^ = 0.892; *p* ≤ 0.001). On day 21 posttreatment, thermal withdrawal latency correlated positively with the BDNF gene expression (*r*^2^ = 0.071; *p* = 0.01).

## 4. Discussion

The current research is the first to shed light on the effects of pregabalin-ADMSC combined treatment compared to pregabalin or ADMSC monotherapy to treat neuropathic pain in a rodent model of sciatic nerve crush injury. We found that (1) sciatic nerve injury increased thermal hyperalgesia, decreased medial gastrocnemius contraction force (i.e., EMG amplitude) and nerve conduction velocity, distorted the gait, and showed an increase in the BDNF expression and inflammatory cytokines. (2) Combined treatment of pregabalin and ADMSC effects was comparable to ADMSC alone to improve neuropathic pain studied parameters.

Weekly thermal withdrawal latency assessments showed the rapid, potent antihyperalgesic effect of ADMSCs. On the other hand, pregabalin showed a significant, albeit delayed, and less potent antihyperalgesic effect than ADMSCs or combined treatments. These results are concordant with studies using ADMSCs in a neuropathic pain mouse model of sciatic nerve chronic constriction injury [20, 21].

ADMSCs or combined treatments improved walking abilities, as depicted by SFI, in the animal model of neuropathic pain. Our results are concordant with others using ADMSCs [22–25] or pregabalin for treating nerve injuries, though the latter's role remains controversial [22, 26]. We found a robust positive correlation between SFI and the levels of IL1-*β* in the sciatic nerve on day 1 postinjury, highlighting the role of inflammation in the functional damage incurred to the sciatic nerve.

All treatments improved the electrophysiological measures compared to the injured nontreated group. All treatments significantly increased the sciatic nerve conduction velocity and increased the amplitude of EMG of the gastrocnemius muscle, consistent with other reports [27, 28], suggesting regeneration of the transected nerve. Pregabalin was reported to significantly increase motor nerve conduction velocity in a partial sciatic nerve ligation rat model [29].

Our findings that sciatic nerve injury increased BDNF gene expression, and that all treatments increased further, especially the combined treatment, agree with other reports [30–32]. Pregabalin was reported to increase serum BDNF levels in a recent randomized, double-blind, controlled study on patients with thoracic postherpetic neuralgia [33]. The role of BDNF in the alleviation of hyperalgesia was emphasized as the BDNF gene expression positively correlated with thermal withdrawal latency on day 21 posttreatment.

The unbalanced response of the anti-inflammatory cytokines can lead to and maintain the pain [34]. All treatments decreased the nerve tissues and serum levels of the proinflammatory cytokines (IL-1*β* and TNF-*α*) in comparison to the injured group. This decrease was more pronounced in the ADMSC groups. This interesting rapid and potent antihyperalgesic effect of ADMSCs, over pregabalin, may be attributed to the ability of stem cells themselves to secrete and synthesize the cytokines that enhance their migration toward damaged tissues [25, 35]. ADMSCs' immunomodulatory role is thought to occur directly by cellular contact or indirectly by releasing specific factors such as tumor growth factor-beta and IL-10 or suppressing the T-cells and the inflammatory response [36]. Indeed, ADMSCs diminished the hyperalgesia and allodynia and normalized the inflammatory response in a murine model of sciatic nerve constriction injury (i.e., neuropathic pain model) [12].

All treatments decreased the nerve tissues and serum levels of IL-10, suggesting a relevant role of IL-10 in regulating sensory hypersensitivity. These results are in accordance with other reports using ADMSCs to blunt neuroinflammation [12, 36] and correct the balance between proinflammatory and anti-inflammatory cytokines. This effect was maintained for up to 12 weeks after treatment [36]. Gabapentin, another antiepileptic drug, structurally similar to pregabalin, increased IL-10 and decreased proinflammatory cytokines (TNF-*α*, IL-1*β*, and IL-6) in a rat model of neuropathic pain when injected intrathecally [37].

There are several important factors to consider when evaluating nerve regeneration in response to therapy, such as arrangement, nerve fiber myelin sheath, and Schwan cell conditions. Following peripheral nerve trauma, although the severed axons had an intrinsic capability to regenerate, the rate of axonal regeneration remains a significant clinical problem, especially after severe injuries [22]. We found that all treatments improved nerve regeneration parameters with more improvement in response to ADMSCs and the combined treatments. These results are in accordance with others where ADMSCs were shown to promote axon regeneration and myelin formation, decreased fibrin degradation, and restored nerve architecture in a rat model of neuropathic pain [23, 38]. Unlike ADMSCs, pregabalin treatment effects on histomorphometric nerve parameters remain controversial [22, 25].

We chose a dose of 1 × 10^6^ ADMSCs in rats' tail vein as it has been reported as the effective dose in treating neuropathic pain [38]. We selected the 7^th^ day postinjury for the injection of ADMSCs, as it was the appropriate time to ensure the presence of the highest degree of pain and began therapy at that point [12]. Our study's limitations are its observational nature and the use of thermal withdrawal latency as a single method to test hyperalgesia.

In summary, this study revealed that the combined treatment of stem cells and pregabalin is associated with greater improvement of the sciatic nerve structure and function compared to pregabalin monotherapy in an animal model of neuropathic pain. Further studies on various doses, timing, and duration of combined treatment are warranted.

## Data Availability

Data can be available on request.

## Abbreviations

ADMSCs: Adipose-derived mesenchymal stem cells
BDNF: Brain-derived neurotrophic factor
DMEM: Dulbecco's Modified Eagle's Medium
ELISA: Enzyme-linked immunosorbent assay
IL: Interleukin
MACS: Magnetic associated cell separation columns
PBS: Phosphate-buffered saline
RT-PCR: Real-time polymerase chain reaction
SFI: Sciatic function index
TNF-*α*: Tumor necrosis factor alpha.

## Supplementary Materials

Supplementary Materials Table (S1): serum (S) and nerve (N) cytokine levels posttreatment. Figure S1: research design flowchart. Figure S2: histology (×400) of sciatic nerve sections. Black arrows for nerves, yellow arrows for infiltrate, green arrows for blood vessels, blue arrows for hyaline degenerations, and orange arrows for vacuolar degeneration. (a) Sham section reveals normal nerve arrangement, regularly arranged axons, and unremarkable peri- and endoneurium. The myelinated fibers reveal an intact myelin sheath around the intact nerve fiber. (b) Injured section reveals disorganized axons with marked vacuolar degeneration, the large empty vacuoles in between axons. There is a thickened wall of the blood vessel and an increase in the inflammatory cellular infiltration. Most axons are thinned out, and some are collapsed and transformed into a pink hyaline material. (c) Adipose tissue-derived mesenchymal stem cell (ADMSCs) section showing a moderately restored regular arrangement of the axons and increased thickness and regularity of the nerve. The vessels are thin-walled. Epineurium is thin and regular; there are very few foci showing minimal vacuolar degeneration of axons. (d) Pregabalin section shows mildly restored regular arrangement of the axons and the regularity of the nerve, with still some vacuolar degeneration and some hyaline degeneration and thickness in the vascular wall. (e, f) Combined section shows a strong restoration of the regular arrangement of the axons and the regularity of the nerve, and normal epineurium, and the vessels are thin-walled.
