# Supplementary material for: Combined Treatment of Adipose Derived-Mesenchymal Stem Cells and Pregabalin Is Superior to Monotherapy for the Treatment of Neuropathic Pain in Rats
Source: Stem Cells Int. 2021 Feb 15;2021:8847110. doi: 10.1155/2021/8847110 (PMC7899775; doi:10.1155/2021/8847110)

**TABLE (S1): Serum (S) and Nerve (N) Cytokine levels Post-treatment**.

| **Cytokine** | **Day** | **Sham** | **Injured** | **ADMSCs** | **Pregabalin** | **Combined** |
| --- | --- | --- | --- | --- | --- | --- |
| **IL1-1B (N)** | 1 | 38.1 ± 6.49 | 65 ±0 .29 * | 45.4 ± 4.28 # | 50±2.3 $ | 42.3 ± 2.4 # |
|  | 7 | 57 ± 11 | 90 ± 6.8 ** | 47.1 ± 4 ## | 60 ±2.8 ## | 45 ± 3.4 ##** |
| **IL1-B (S)** | 1 | 77.9 ± 3.9 | 167 ± 3.9 * | 145 ± 3.5 # | 59±6.5$@ | 141 ± 5.5# |
|  | 7 | 47 ± 5.1 | 74 ± 2.6 ** | 53 ± 3.5 ## | 59 ± 6.5 ##$$@@ | 52 ± 2 ## |
| **TNF-a (N)** | 1 | 40 ± 5.6 | 77 ± 5.8 * | 40 ± 3.8 # | 55±3.4 $@ | 44.3 ± 4.2 # |
|  | 7 | 52 ± 5.3 | 100 ± 8.8 ** | 55 ± 2.5 ## | 69.7 ±6.9 ##$$@@ | 53 ± 2.7 ## |
| **TNF-a (S)** | 1 | 30 ± 2.2 | 61.5 ± 6.6 * | 45 ± 2.9 # | 60±5.6 $@ | 47 ± 3.4 # |
|  | 7 | 25 ± 2.9 | 77 ± 4.1 ** | 50 ± 6.1 ## | 65 ±3.9 ##$$@@ | 51 ± 57 ## |
| **IL-10 (N)** | 1 | 150 ± 9.3 | 24 ± 4.4 * | 32±4.2 | 29±2.2 | 35 ± 4.5 #* |
|  | 7 | 148 ± 5.7 | 27 ± 4.2 ** | 300 ± 19 ## | 250 ±15.8 ##$$@@ | 354 ± 8.9 ##** |
| **IL-10 (S)** | 1 | 52 ± 5.34 | 53 ± 3.4 | 67 ± 3.5 # | 60 ±5.6 # | 68 ± 3.7 #* |
|  | 7 | 54 ± 4.2 | 55 ± 3.2 | 53 ± 3.2 | 55 ±4.5 | 54.7 ± 5.1 |

Data are shown as mean ± SEM – Cytokine level in pg/mL.

* significant when compared to the sham group on Day 1, and ** on Day 7.

# significant when compared to the injured group on Day 1 and ## on Day 7.

$ significant when compared to the combined group on Day 1, and $$ on Day 7.

@ significant when compared to ASCs group in 1 day and @@ on Day 7.

**Figure S1. Research Design Flowchart.**


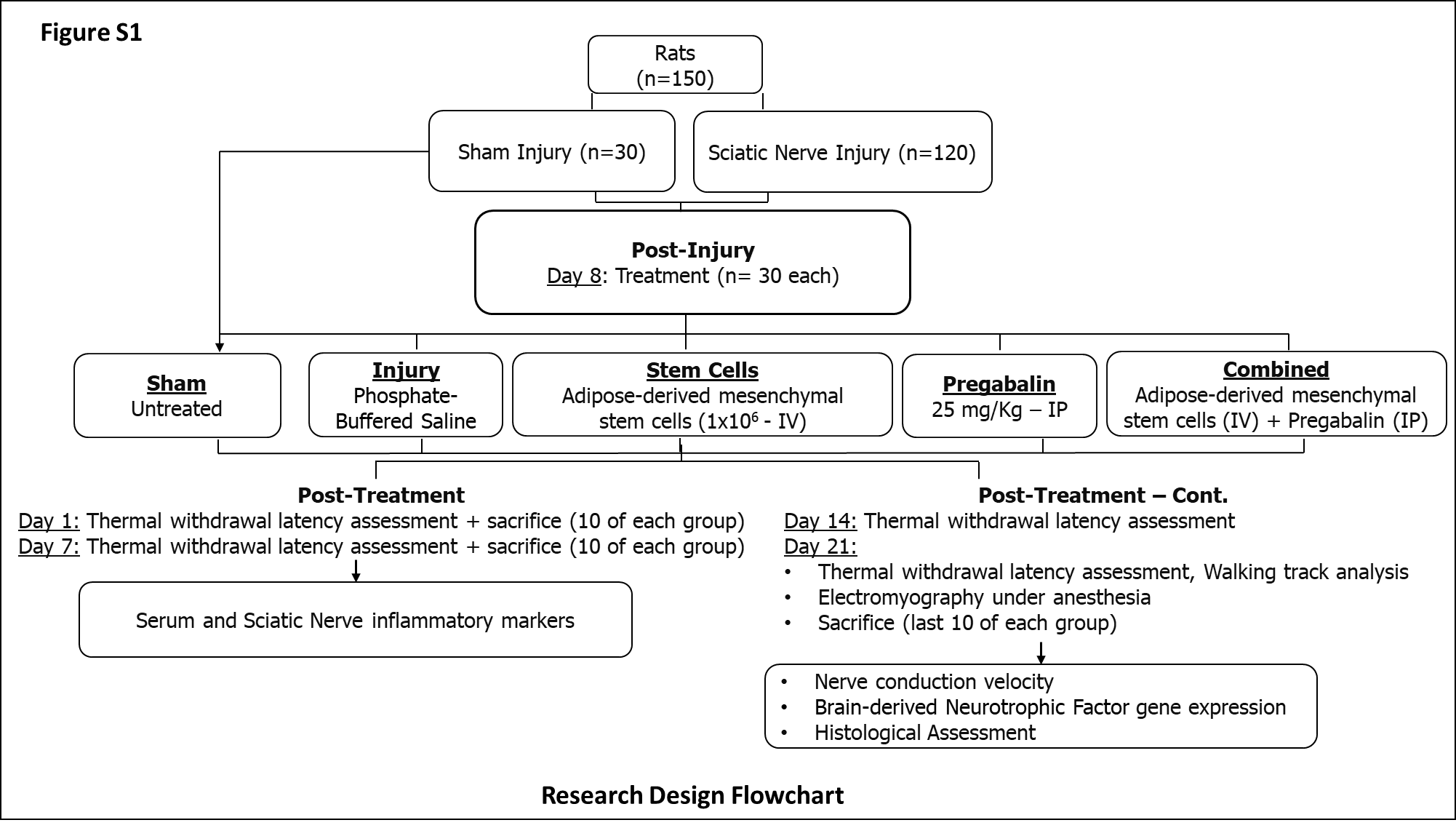


**Figure S2. Histology (X400) of sciatic nerve sections.**
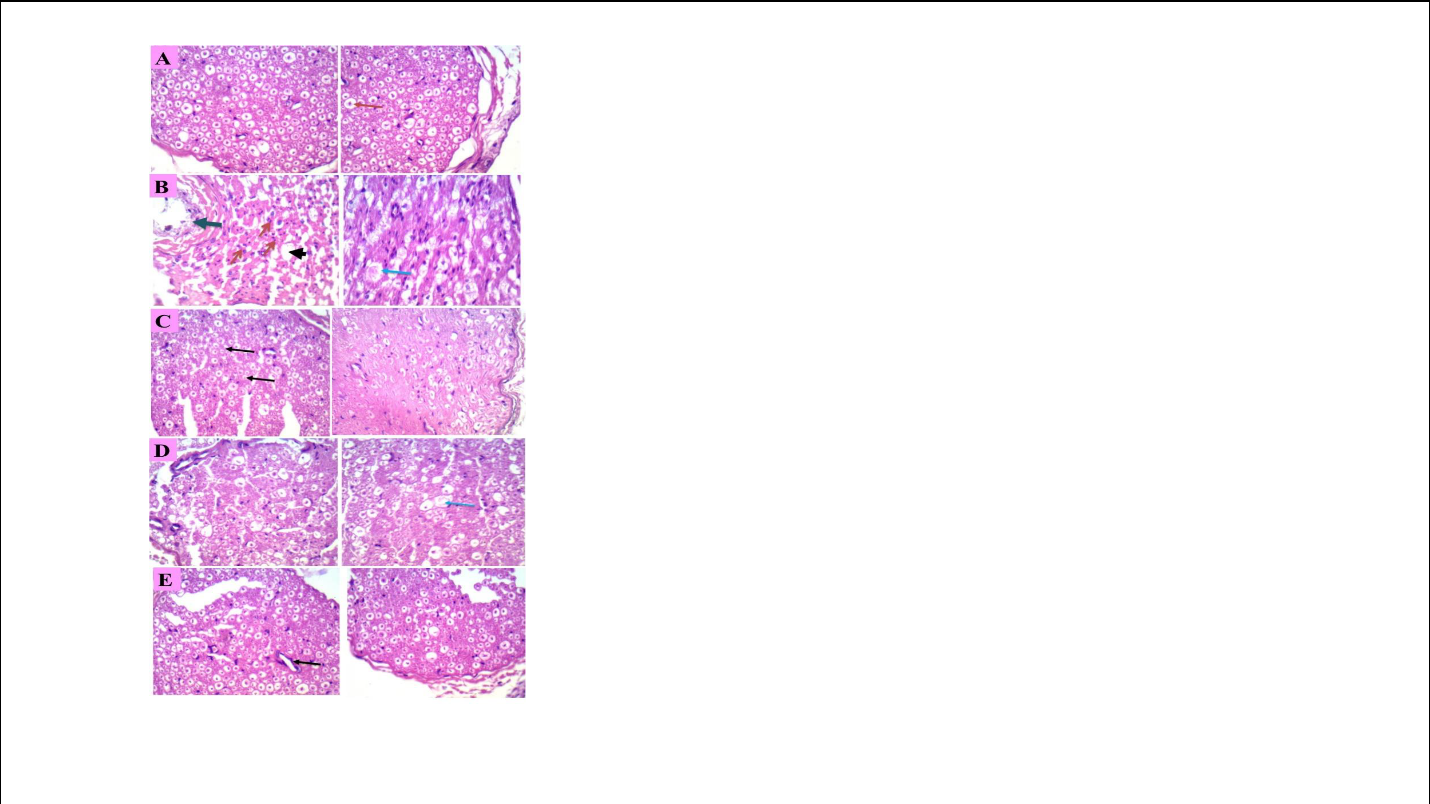

Supplement: Supplementary Materials — Table (S1): serum (S) and nerve (N) cytokine levels posttreatment. Figure S1: research design flowchart. Figure S2: histology (×400) of sciatic nerve sections. Black arrows for nerves, yellow arrows for infiltrate, green arrows for blood vessels, blue arrows for hyaline degenerations, and orange arrows for vacuolar degeneration. (a) Sham section reveals normal nerve arrangement, regularly arranged axons, and unremarkable peri- and endoneurium. The myelinated fibers reveal an intact myelin sheath around the intact nerve fiber. (b) Injured section reveals disorganized axons with marked vacuolar degeneration, the large empty vacuoles in between axons. There is a thickened wall of the blood vessel and an increase in the inflammatory cellular infiltration. Most axons are thinned out, and some are collapsed and transformed into a pink hyaline material. (c) Adipose tissue-derived mesenchymal stem cell (ADMSCs) section showing a moderately restored regular arrangement of the axons and increased thickness and regularity of the nerve. The vessels are thin-walled. Epineurium is thin and regular; there are very few foci showing minimal vacuolar degeneration of axons. (d) Pregabalin section shows mildly restored regular arrangement of the axons and the regularity of the nerve, with still some vacuolar degeneration and some hyaline degeneration and thickness in the vascular wall. (e, f) Combined section shows a strong restoration of the regular arrangement of the axons and the regularity of the nerve, and normal epineurium, and the vessels are thin-walled. [file 8847110.f1.zip › Table S1.docx]
